# Metabolic programs associated with GDSC2-inferred oxaliplatin sensitivity are favorably prognostic in gastric cancer: transcriptomic and single-cell evidence

**DOI:** 10.3389/fgene.2026.1947421

**Published:** 2026-09-23

**Authors:** Lingyun Wei, Chengnv Li, Cong Wang

**Affiliations:** 1 Department of Anesthesiology, Chun’an County First People’s Hospital, Hangzhou, Zhejiang, China; 2 Department of Rehabilitation Medicine, Xuzhou First People’s Hospital, Xuzhou, Jiangsu, China

**Keywords:** drug sensitivity, gastric cancer, GDSC2, oxaliplatin, transcriptomics

## Abstract

**Background:**

Oxaliplatin is widely used in gastric cancer (GC), yet molecular features associated with sensitivity are heterogeneous and incompletely defined. Pharmacogenomic models trained in cancer cell lines can generate hypotheses about drug-response biology, but their transfer to patient tumors requires cautious interpretation. We, therefore, integrated bulk transcriptomic, pharmacogenomic, prognostic, and single-cell data to characterize transcriptional programs associated with GDSC2-inferred oxaliplatin sensitivity in GC.

**Methods:**

A ridge-regression model trained on 396 GDSC2 cell lines was used to predict oxaliplatin area-above-curve (AAC) from expression data and was transferred to 412 TCGA-STAD primary tumors. Resistant-like and sensitive-like tumors were defined by the lower and upper predicted-AAC quartiles. Differential expression, gene-set enrichment, WGCNA, immune-cell scoring, metabolic-program scoring, and ROC analyses were performed, respectively. Prognostic associations were examined in GSE62254 and GSE26253, and cell-type localization was explored in the GSE134520 single-cell dataset. For experimental corroboration, AGS gastric cancer cells were exposed to oxaliplatin (10–20 μM) for 24 h, followed by qRT-PCR analysis of ALB, LDHA, FASN, and CPT1A mRNA expression relative to untreated AGS controls.

**Results:**

Cross-validation of the GDSC2 ridge model showed moderate agreement between predicted and observed oxaliplatin AAC (Pearson r = 0.50; Spearman rho = 0.44). In TCGA-STAD, resistant-like tumors showed higher glycolysis scores (Mann–Whitney *p* = 2.5e−11; Cohen d = 0.94), and glycolysis discriminated resistant-like from sensitive-like tumors with an AUC of 0.77. Oxidative phosphorylation was strongly enriched in resistant-like tumors (NES = +2.98), whereas adaptive immune scores were relatively lower and myeloid/neutrophil scores were higher. Importantly, metabolic programs associated with inferred resistance were not uniformly associated with poor prognosis: higher glycolysis, oxidative-phosphorylation, and fatty-acid-oxidation program expression showed favorable or neutral survival associations in the independent cohorts. Single-cell analysis localized glycolytic activity across multiple gastric cell types, with the highest median score in stromal cells. In oxaliplatin-treated AGS cells, qRT-PCR showed lower ALB and higher LDHA, FASN, and CPT1A mRNA expression than in untreated controls (all reported *p* < 0.001).

**Conclusion:**

Gastric tumors classified as oxaliplatin resistant-like by a GDSC2-derived transfer model exhibit coordinated metabolic and immune transcriptional features, particularly increased glycolytic and oxidative programs. However, these findings describe model-inferred drug sensitivity rather than clinically observed oxaliplatin resistance, and the favorable prognostic associations of several resistance-associated metabolic programs emphasize the distinction between drug-sensitivity inference and prognosis. The AGS cell qRT-PCR experiment provides limited mRNA-level experimental corroboration for selected metabolic genes under oxaliplatin exposure but does not establish protein-level or causal mechanisms. Direct validation in clinically annotated oxaliplatin-treated cohorts and additional functional experiments are required.

## 1 Introduction

Gastric cancer (GC) remains a major global health burden, with more than one million new cases and approximately 769,000 deaths estimated worldwide in 2020 (Sung et al., 2021). Platinum-fluoropyrimidine regimens are central to the systemic treatment of advanced GC, and oxaliplatin is widely used in contemporary chemotherapy combinations (Smyth et al., 2020). Nevertheless, intrinsic and acquired oxaliplatin resistance limits treatment durability. Experimental studies have identified diverse resistance mechanisms in GC, including DNA-repair regulation and MAPK-associated transcriptional programs, underscoring that oxaliplatin response is biologically heterogeneous rather than governed by a single pathway (Fang et al., 2022; Li et al., 2023).

Large pharmacogenomic resources provide an opportunity to study this heterogeneity systematically. The Genomics of Drug Sensitivity in Cancer (GDSC) links molecular profiles of cancer cell lines with quantitative drug-response phenotypes and has enabled machine-learning approaches to infer pharmacogenomic associations (Iorio et al., 2016). However, transferring a model trained in heterogeneous cancer cell lines to primary tumors is inherently exploratory because tissue lineage, platform effects, tumor microenvironment, and clinical drug exposure are not equivalent. Accordingly, predicted drug sensitivity in a tumor transcriptome should be interpreted as a model-derived molecular phenotype rather than as a substitute for observed clinical response.

Metabolic state is a plausible component of drug-response heterogeneity. Tumor glycolysis, oxidative metabolism, and lipid utilization can alter redox balance, biosynthetic capacity, and nutrient competition within the tumor microenvironment. These metabolic processes can also shape immune-cell function: glucose competition may constrain effector lymphocytes, and tumor-derived lactate can influence macrophage polarization and other immune states (Chang et al., 2015; Colegio et al., 2014; Ippolito et al., 2019). Such relationships are context-dependent, however, and transcriptomic co-variation alone cannot establish a causal mechanism linking metabolism, immune composition, and chemotherapy resistance.

Several public GC resources enable these questions to be examined across complementary biological scales. TCGA-STAD provides bulk tumor RNA-seq and molecular annotations (Cancer Genome Atlas Research Network, 2014); GSE62254 provides an independently characterized ACRG cohort with survival and molecular subtype information (Cristescu et al., 2015); GSE26253 contains 432 FFPE gastric adenocarcinomas with recurrence follow-up after curative surgery and adjuvant chemoradiotherapy (Lee et al., 2014); and GSE134520 provides a single-cell atlas spanning gastritis, intestinal metaplasia, and early GC (Zhang et al., 2019). Together, these datasets allow drug-sensitivity-associated transcriptional programs to be placed in prognostic, molecular-subtype, and cell-type contexts without conflating those contexts with direct clinical oxaliplatin-response validation.

In this study, we trained a ridge regression model on GDSC2 oxaliplatin response and applied it to TCGA-STAD tumors to derive an explicitly model-inferred sensitivity phenotype. We then characterized associated differential-expression, pathway, co-expression, immune, and metabolic features; examined their prognostic behavior in GSE62254 and GSE26253; localized selected programs in GSE134520 single-cell data; and performed limited *in vitro* qRT-PCR validation in AGS gastric cancer cells exposed to oxaliplatin (10–20 μM, 24 h). The study is designed as a hypothesis-generating integrative analysis and does not claim direct clinical validation of oxaliplatin resistance or immunotherapy response.

## 2 Methods

### 2.1 Data sources

Bulk transcriptomes were obtained from The Cancer Genome Atlas stomach adenocarcinoma project (TCGA-STAD): STAR-based gene-level counts for 448 samples (412 primary tumors and 36 solid-tissue normals; 415 patients), together with harmonized clinical annotations, including AJCC pathologic stage, Lauren classification, MSI, and EBV status. Two independent gastric cancer microarray cohorts served as prognostic validation: GSE62254 (ACRG cohort, 300 primary tumors, Affymetrix HG-U133 Plus 2.0, with overall survival and ACRG molecular subtypes; Cristescu et al., 2015) and GSE26253 (432 FFPE gastric adenocarcinomas, Illumina HumanRef-8 WG-DASL v3.0, with recurrence-free survival; Lee et al., 2014). Single-cell RNA-seq data from 13 gastric antral biopsies across the gastritis–intestinal metaplasia–early gastric cancer cascade (GSE134520, 10x Genomics; Zhang et al., 2019) were used for cell-type-level localization. Cell-line pharmacogenomic data were obtained from the Genomics of Drug Sensitivity in Cancer (GDSC2) resource (ORCESTRA PharmacoSet v2-8.2) (Cancer Genome Atlas Research Network, 2014; Cristescu et al., 2015; Lee et al., 2014; Zhang et al., 2019).

### 2.2 Expression preprocessing

Raw integer counts were retained for model-based analysis. For visualization and linear modeling, variance-stabilizing transformation (DESeq2 vst) was applied; 18,511 genes passing a counts ≥ 10 were included in differential analyses. Tumor-versus-normal differential expression was analyzed using DESeq2 (Wald test, BH adjustment). Microarray cohorts were used in their published normalized (log2) space; probe-to-gene mapping used the platform annotation tables, and duplicate probes were median-collapsed (Love et al., 2014).

### 2.3 Inference of oxaliplatin sensitivity and its scope

A ridge-regression model was trained to predict the GDSC2 area-above-curve (AAC) for oxaliplatin from cell-line expression (U133A platform, 11,808 gene-level features). Cell lines were joined between the sensitivity and expression tables using the PharmacoSet-internal cell identifiers (cellid), which recovered substantially more matched lines than name-based matching (396 oxaliplatin lines versus 309 by exact name matching). Model performance was evaluated by 5-fold cross-validation, and the fitted model was applied to the 412 TCGA-STAD primary tumors after per-gene standardization using the training-set mean and standard deviation; tumors in the bottom quartile of predicted AAC were labeled resistant-like, and the top quartile sensitive-like. Four scope limitations apply. First, gastric-origin cell lines constitute a small minority of the training set (the exact count varies by annotation field), and the pan-cancer training set additionally contains esophageal lines and at least one misclassified line (RF-48, lymphoma), so the model is a pan-cancer transfer rather than a gastric-specific predictor. Second, the model was trained on Affymetrix U133A microarray expression and applied to TCGA RNA-seq vst values without cross-platform calibration. Third, no cohort used here carries clinical oxaliplatin response or resistance labels; the resistant/sensitive contrast is, therefore, model-inferred, and GSE62254, GSE26253, and GSE134520 serve as prognostic or descriptive cohorts, not as drug-response validation. Fourth, because the resistant-like and sensitive-like groups are derived from the same expression matrix that is subsequently analyzed, all differential expression, enrichment, co-expression, and discrimination analyses describe the transcriptomic features of the model output; they are not independent validations of drug resistance. All sensitivity-related statements below refer to this inferred quantity.

### 2.4 Differential expression, enrichment, and co-expression

Differential expression between resistant-like and sensitive-like tumors was assessed using limma on the vst matrix (Benjamini–Hochberg FDR). Gene-set enrichment analysis was performed using GSEA (clusterProfiler gseGO, biological process ontology). Signed co-expression network analysis was performed using WGCNA (soft power 6, signed network, top 5,000 variable genes, and minimum module size 30). Module-trait associations were quantified using Pearson correlation (the WGCNA default); the eigengene scatter panels additionally report Spearman rank correlations, and both coefficients are labeled as such in the figures (Langfelder and Horvath, 2008; Ritchie et al., 2015; Yu et al., 2012; Subramanian et al., 2005).

### 2.5 Program scoring and discrimination analysis

Immune cell scores were computed as the mean expression of curated LM22-derived marker gene subsets (Newman et al., 2015) normalized to proportions. Metabolic program scores (glycolysis, oxidative phosphorylation, drug efflux, and carnitine/fatty-acid oxidation) were computed as the mean expression of curated pathway gene sets (HALLMARK/KEGG/GO-derived; gene lists fixed before analysis). The discriminative value of each program for the inferred resistant-versus-sensitive contrast was quantified by ROC AUC with permutation-based significance, and group comparisons used Mann–Whitney or Kruskal–Wallis tests. In GSE62254, survival coding was verified against the original clinical follow-up table: OS_censor = 0 denotes death (152 events) and OS_censor = 1 denotes censored survivors (148); event status was reconstructed accordingly. Programs were tested for associations with survival using Cox proportional hazard models (per standard-deviation increase, with and without stage adjustment) and Kaplan–Meier curves using the log-rank test. In GSE26253, recurrence status was taken from the GEO annotation (1 = recurrence, 177 events); associations in this cohort are reported at nominal significance without correction for the four programs tested (Newman et al., 2015).

### 2.6 Single-cell analysis

Cells were filtered (200–6,000 detected genes, <20% mitochondrial reads; 43,992 cells retained), library-size was normalized, and log1p values were transformed. Highly variable genes (n = 2,000) drove PCA, neighborhood graph, UMAP, and Leiden clustering; cell types were assigned by marker-set scoring. Program scores were computed per cell, but all condition-level single-cell comparisons are descriptive and anchored at the sample level: the EGC condition comprises a single biopsy (n = 1), so no cell-level or sample-level statistical inference is performed for EGC contrasts (Wolf et al., 2018).

### 2.7 qRT-PCR validation

AGS human gastric adenocarcinoma cells were maintained under standard culture conditions. For oxaliplatin exposure, cells were treated with oxaliplatin at 10–20 μM for 24 h; untreated AGS cells cultured in parallel served as controls. Total RNA was extracted using TRIzol reagent, and cDNA was synthesized using PrimeScript RT (Takara Bio, Shiga, Japan; RR037A) Quantitative real-time PCR was performed on a StepOnePlus Real-Time PCR System (Applied Biosystems, Foster City, CA, United States) using SYBR Green chemistry. Relative mRNA expression was normalized to GAPDH and calculated using the 2^−ΔΔCt method. Primer sequences (5′→3′) were as follows: ALB-F, GCC​TGA​ACT​GTG​CGG​AGA​AG; ALB-R, TCT​TGG​CAG​CAG​CAC​CTT​CT; LDHA-F, ATG​GCA​ACT​CTA​AAG​GAT​CAG​C; LDHA-R, CCA​ACC​CCA​ACA​ACT​GTA​ATC​T; FASN-F, AGC​GGG​TCA​TGA​CTC​CCA​AT; FASN-R, CAG​CTC​CAT​GAT​GCA​AGG​GAT; CPT1A-F, ATC​CCT​TGT​CAC​ATC​CGC​T; CPT1A-R, GCA​GCT​TCA​GGG​TAG​CTG​TT; GAPDH-F, GTC​TCC​TCT​GAC​TTC​AAC​AGC​G; GAPDH-R, ACC​ACC​CTG​TTG​CTG​TAG​CCA​A. For each target gene, oxaliplatin-treated and control groups were compared using a two-tailed Student’s t-test, with Bonferroni correction across the four target genes. Adjusted *p* < 0.05 was considered statistically significant.

### 2.8 Statistical software

Analyses used R 4.4.3 (DESeq2, limma, clusterProfiler, WGCNA, survival, and glmnet) and Python 3 (scanpy, scikit-learn, lifelines, pandas, and scipy). All *p*-values are two-sided where applicable; multiple-testing correction is stated per analysis.

## 3 Results

### 3.1 A transcriptome-trained model infers oxaliplatin sensitivity in gastric tumors

The TCGA-STAD cohort composition, expression-count distribution, and tumor-versus-normal PCA are summarized in Figures 1A–C. We trained a ridge model on 396 GDSC2 cell lines (joined to expression by PharmacoSet-internal identifiers) to predict oxaliplatin AAC from gene expression. Five-fold cross-validation showed significant agreement between predicted and observed sensitivity (Pearson r = 0.50, *p* = 3.9e−26; Spearman rho = 0.44, *p* = 1.5e−20; Figure 1D). Applying the model to 412 TCGA-STAD primary tumors yielded a continuous sensitivity spectrum, from which we defined 103 resistant-like and 103 sensitive-like tumors at the quartile extremes (Figure 1E). The post-quality-control single-cell cohort comprised 43,992 cells across five gastric conditions (Figure 1F). This quantity is model-inferred (no clinical response labels exist in any cohort used here; gastric-origin lines are a small minority of the training set, and the U133A-trained model was applied to RNA-seq vst without cross-platform calibration; see Methods).

**FIGURE 1:**
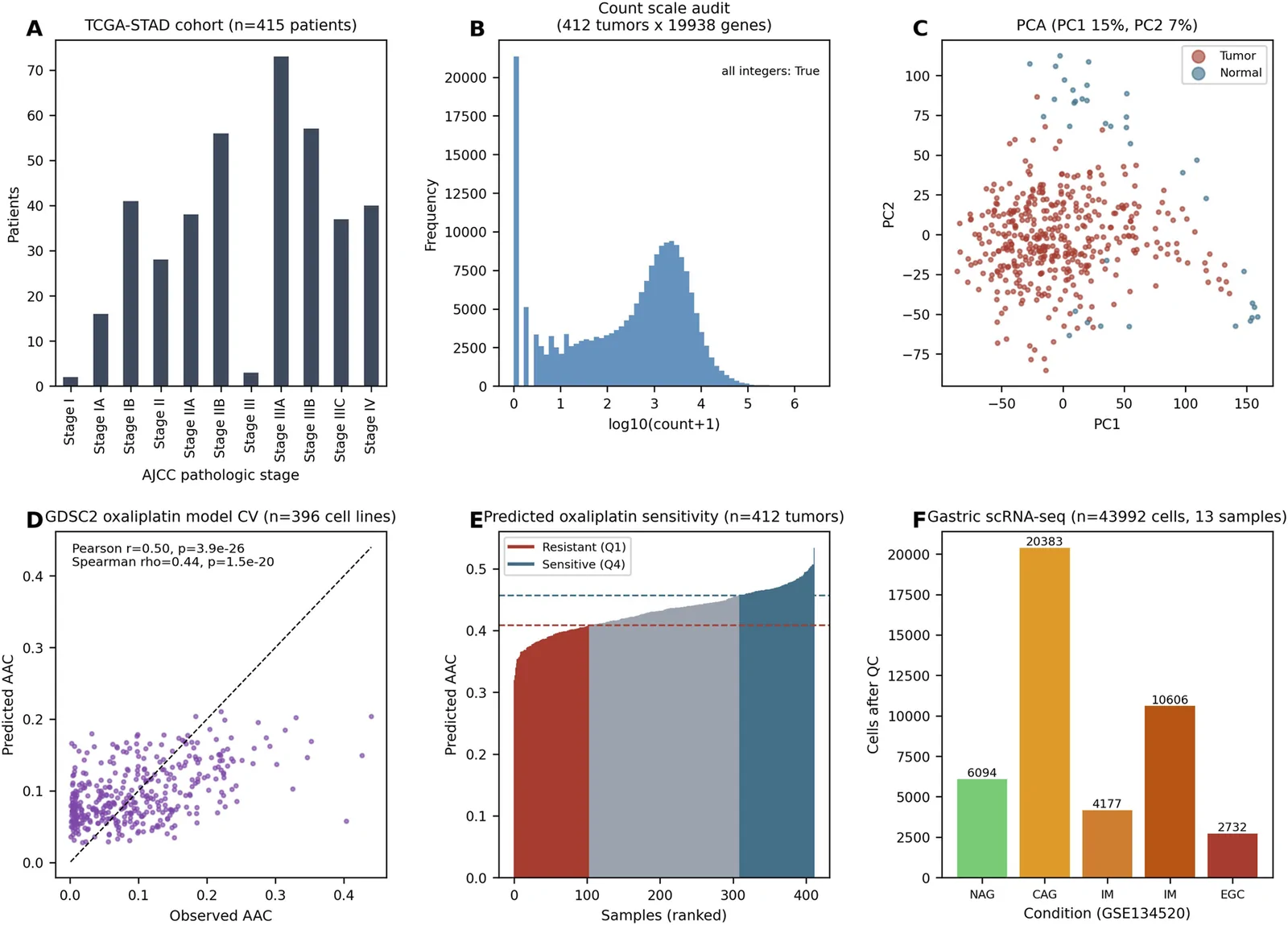
Study cohorts, data quality, and transfer modeling of oxaliplatin sensitivity. **(A)** TCGA-STAD cohort composition by AJCC pathologic stage (n = 415 patients). **(B)** Expression scale audit of the STAR count matrix (412 tumors × 19,938 genes); all values are integer, with the distribution shown on a log10(count+1) scale. **(C)** Principal component analysis of tumor (n = 412, red) versus solid-tissue normal (n = 36, blue) samples; PC1/PC2 variance labeled. **(D)** Cross-validated performance of the GDSC2-trained ridge model for oxaliplatin (n = 396 cell lines joined by PharmacoSet cellid; Pearson r and Spearman rho with p values inset). **(E)** Predicted oxaliplatin AAC for 412 TCGA-STAD tumors, ranked; red/blue dashed lines mark the quartile cutoffs defining resistant-like and sensitive-like groups. **(F)** Cell counts per condition after QC in the gastric single-cell dataset GSE134520 (43,992 cells, 13 samples).

### 3.2 The glycolytic program discriminates resistant-like from sensitive-like tumors

Differential expression between resistant-like and sensitive-like tumors identified 9,558 genes at FDR <0.05 (Figure 2A shows the 2,602 genes additionally exceeding |log_2_FC| > 0.5). Gene set enrichment showed that oxidative phosphorylation and the mitochondrial electron transport chain were the most strongly upregulated programs in resistant-like tumors (oxidative phosphorylation NES = +2.98, adjusted *p* = 5.2e−9; Figure 2B), whereas olfactory sensory terms and antigen-receptor-mediated immune signaling were depleted. The expression patterns of the top 30 differentially expressed genes are shown as a z-scored heatmap in Figure 2C. Among curated programs, glycolysis was the strongest discriminator of the inferred resistant-versus-sensitive contrast: resistant-like tumors carried markedly higher glycolysis scores (Mann-Whitney *p* = 2.5e−11, Cohen d = 0.94; Figure 2D), and the glycolysis score classified the groups with an ROC AUC of 0.77 (permutation *p* = 0.005; Figure 2E). CD4 T-cell, B-cell, and monocyte scores likewise discriminated the groups (AUC = 0.72, 0.69, and 0.65). Among classical platinum-resistance genes, GSTP1 and ERCC1 were upregulated in resistant-like tumors, while ABCC2 was lower, and most others showed no significant difference (Figure 2F).

**FIGURE 2:**
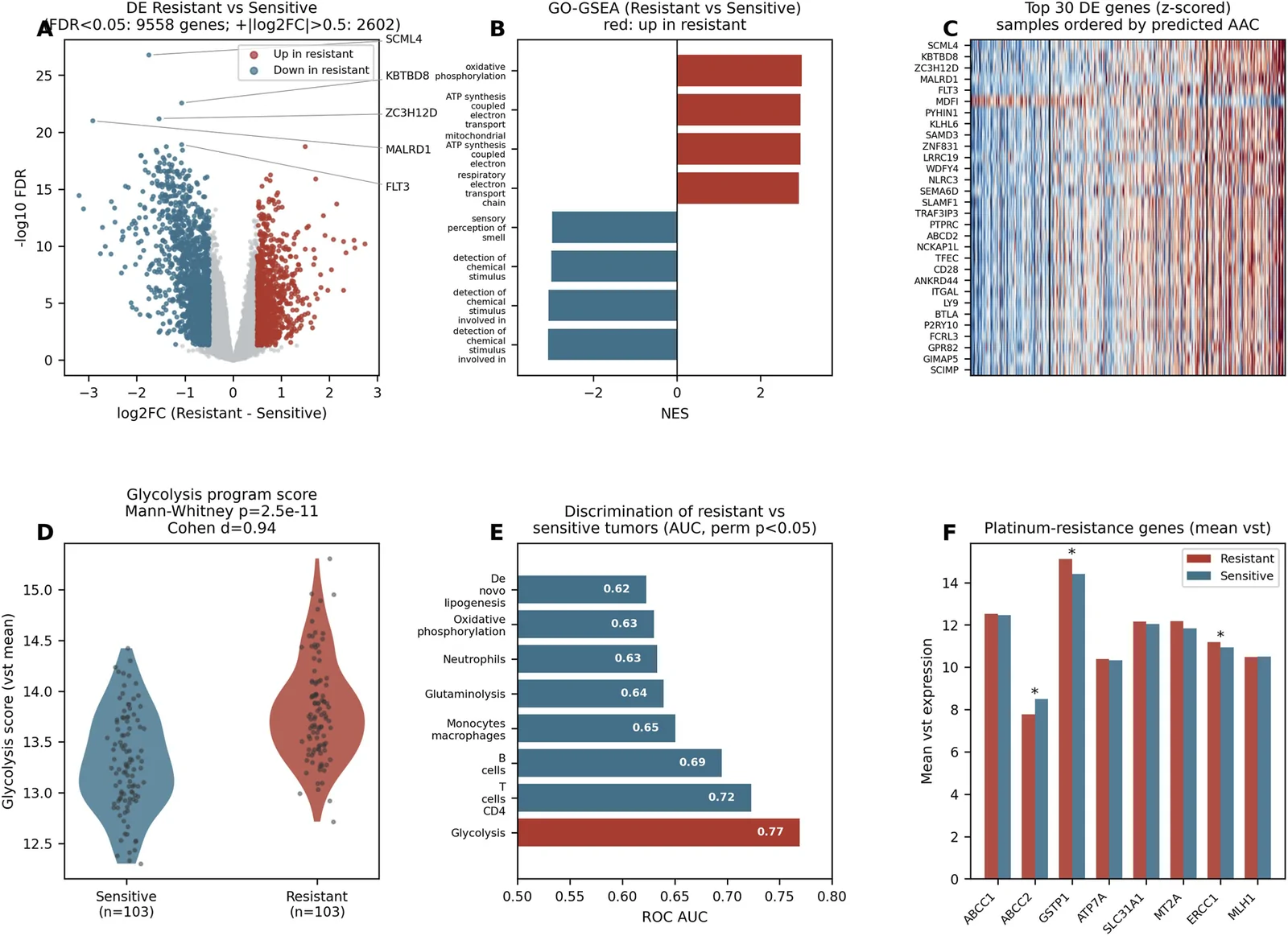
Transcriptomic and pathway features of GDSC2-inferred oxaliplatin resistance. **(A)** Volcano plot of differential expression between resistant-like and sensitive-like tumors; red = up in resistant-like (FDR < 0.05 AND |log2FC| > 0.5), blue = down; the top five genes by FDR are labeled outside the plot with leader lines. **(B)** GO gene-set enrichment (GSEA) of the same comparison; top four positive (red) and top four negative (blue) NES terms; y-axis labels wrapped over up to four lines, unabbreviated. **(C)** Heatmap of the top 30 differential genes (z-scored vst), samples ordered by predicted AAC; vertical lines mark quartile cutoffs. **(D)** Glycolysis program score distributions in sensitive-like (blue) and resistant-like (red) tumors (violin + jittered points); Mann–Whitney *p* and Cohen d annotated. **(E)** ROC AUC of the top 8 significant features discriminating resistant-like versus sensitive-like tumors (descending AUC; permutation *p* < 0.05); AUC values are shown inside the bars; labels are wrapped to two lines. **(F)** Mean expression of classical platinum-resistance genes in resistant-like (red) versus sensitive-like (blue) tumors; asterisks mark *p* < 0.05; gene names rotated 45°.

### 3.3 A squamous differentiation module co-varies with inferred sensitivity

WGCNA of the 5,000 most variable genes identified eight co-expression modules (Figures 3A,B). Module ME6 was the module most strongly associated with lower predicted sensitivity (Pearson r = –0.30, *p* = 3.1e–10; Figure 3C). Its eigengene was negatively associated with predicted AAC (Spearman rho = –0.36, *p* = 2.0e–14; Figure 3D). Conversely, the ME4 eigengene was positively associated with predicted AAC (Spearman rho = +0.36, *p* = 4.7e–14; Figure 3E). The ME6 hub genes (IVL, KRT78, KRT6C, and TMPRSS11 family) mark a squamous/epithelial differentiation program (Figure 3F). These module assignments reflect lineage-related transcriptional states accompanying, rather than proving, drug sensitivity differences.

**FIGURE 3:**
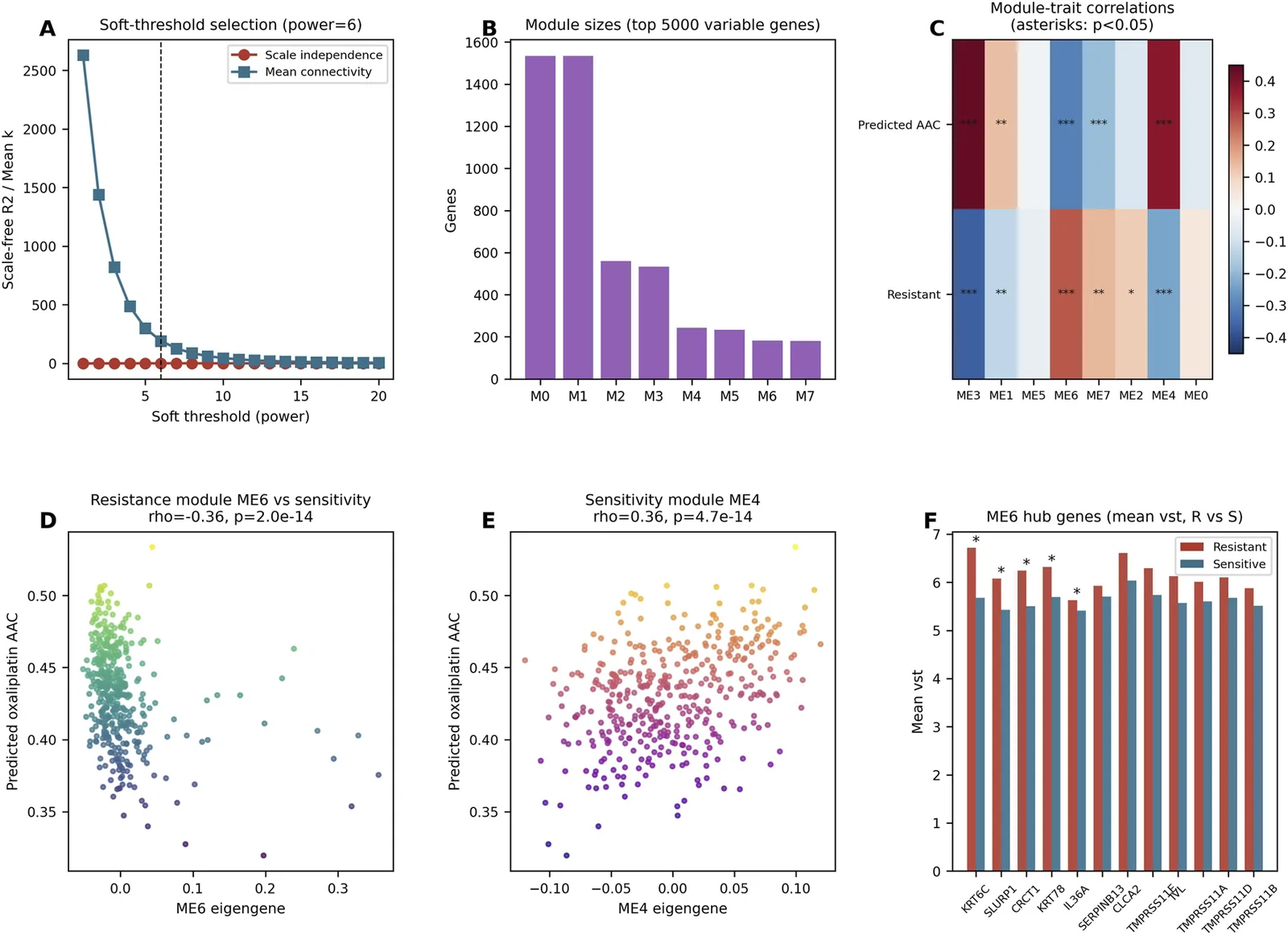
WGCNA co-expression modules associated with predicted oxaliplatin sensitivity. **(A)** WGCNA scale-free topology analysis; soft threshold six selected. **(B)** Module sizes of the eight identified co-expression modules (top 5,000 variable genes). **(C)** Module eigengene-trait correlations (Pearson r, WGCNA default) with predicted AAC and resistance status; asterisks mark *p* < 0.05; module names from the WGCNA output, unmodified. **(D)** Resistance module ME6 eigengene versus predicted AAC (Spearman rho and p). **(E)** Sensitivity module ME4 eigengene versus predicted AAC (Spearman rho and p). **(F)** Mean vst expression of the top ME6 hub genes in resistant-like versus sensitive-like tumors; asterisks mark *p* < 0.05.

### 3.4 Inferred resistance associates with an immune-cold microenvironment

The normalized immune-cell composition across samples ordered by predicted AAC is shown in Figure 4A. Marker-based immune deconvolution showed that resistant-like tumors carried higher myeloid and neutrophil scores, while CD4 T-cell, B-cell, and NK-cell scores tracked sensitivity: CD4 T-cell scores correlated positively with predicted AAC (rho = +0.29, *p* = 3.6e−9) and discriminated resistant-like from sensitive-like tumors (AUC = 0.72; Figures 4B,E). Monocyte/macrophage and neutrophil scores were higher in resistant-like than in sensitive-like tumors (Figure 4C), and checkpoint gene expression showed a graded pattern across the sensitivity spectrum (Figure 4D). The log10-transformed CD8/myeloid score ratio also correlated positively with predicted AAC ( rho = +0.18, *p* = 2.4e−4; Figure 4F).

**FIGURE 4:**
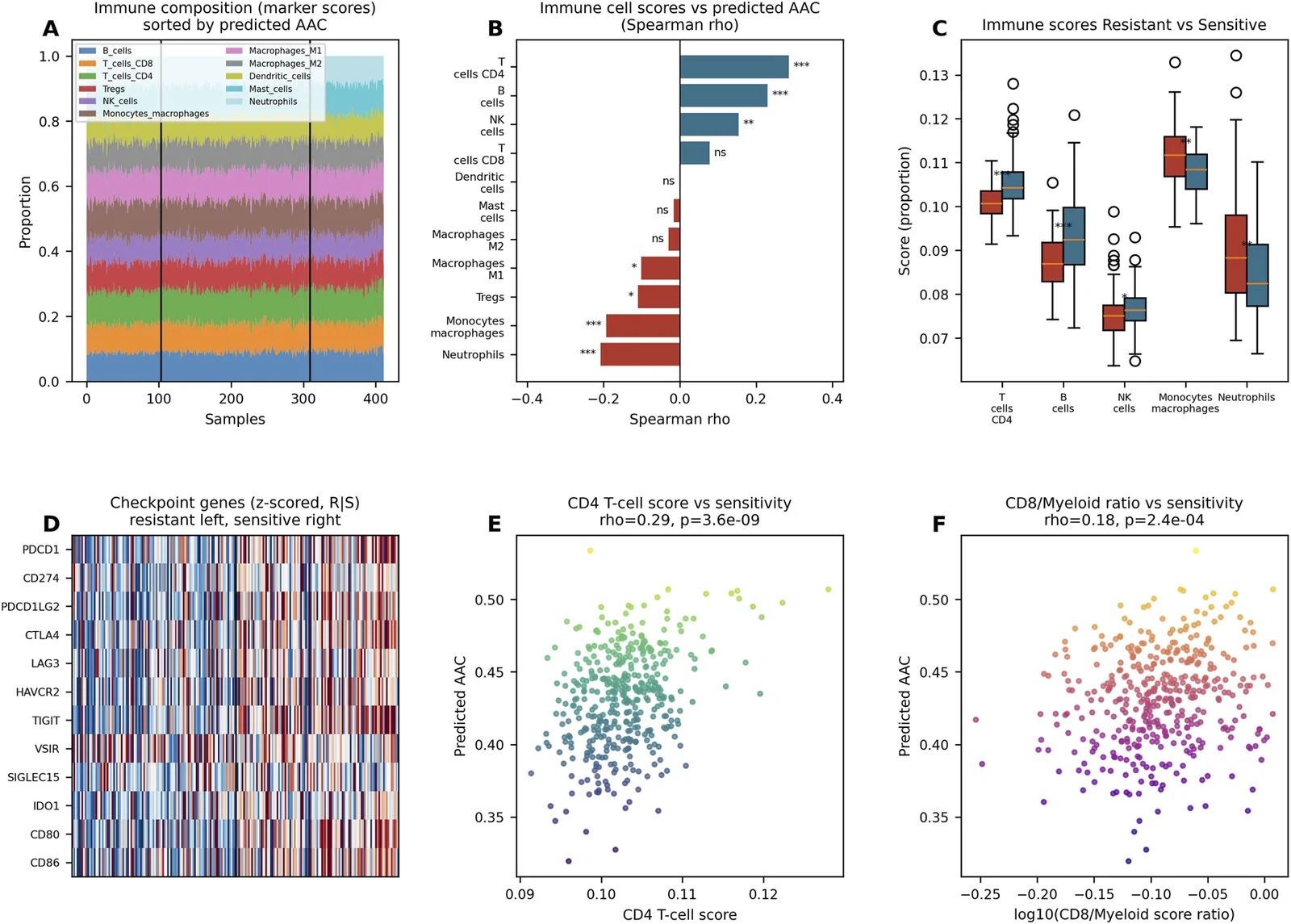
Immune microenvironment features across the inferred oxaliplatin-sensitivity spectrum. **(A)** Stacked immune composition (marker scores normalized to proportions), samples ordered by predicted AAC; quartile cutoffs marked. **(B)** Spearman correlations of immune cell scores with predicted AAC; significance stars placed adjacent to each bar (positional pairing); labels wrapped to two lines. **(C)** Immune cell scores in resistant-like versus sensitive-like tumors for the significant cell types (box plots; Mann-Whitney significance). **(D)** Checkpoint gene expression (z-scored) across the sensitivity spectrum (resistant-like left, sensitive-like right). **(E)** CD4 T-cell score versus predicted AAC. **(F)** log10 CD8/myeloid score ratio versus predicted AAC.

### 3.5 Sensitivity-associated metabolic programs are favorably prognostic in independent cohorts

Glycolysis-score distributions across ACRG molecular subtypes are shown in Figure 5A. Using the verified survival coding for GSE62254 (152 deaths, 148 censored), high glycolysis-program expression was associated with BETTER overall survival (per-standard-deviation Cox HR = 0.77, *p* = 0.0013; median-split log-rank *p* = 0.033; Figures 5B,C), and the association persisted after stage adjustment (HR = 0.84 per SD, *p* = 0.018; Figure 5C) with a quartile dose relationship (log-rank *p* = 0.011; Figure 5D). Glycolysis scores across pathologic stages are shown in Figure 5E. The OXPHOS program showed the same favorable direction (per-SD HR = 0.65, *p* = 3.5e-7), as did the carnitine/FAO program (per-SD HR = 0.84, *p* = 0.040); the drug-efflux program was not prognostic (per-SD HR = 1.02, *p* = 0.78). In the second independent cohort (GSE26253, n = 432, 177 recurrences), higher OXPHOS expression was nominally associated with fewer recurrences (HR = 0.73, *p* = 0.042, uncorrected for the four programs tested; Figure 5F), while glycolysis was not associated (*p* = 0.565). Because GSE26253 is a post-surgery chemoradiotherapy cohort and not an oxaliplatin-response cohort, this association is interpreted as a general RFS association only. Overall, the metabolic programs that mark GDSC2-inferred resistance are associated with favorable, not adverse, prognosis in these cohorts.

**FIGURE 5:**
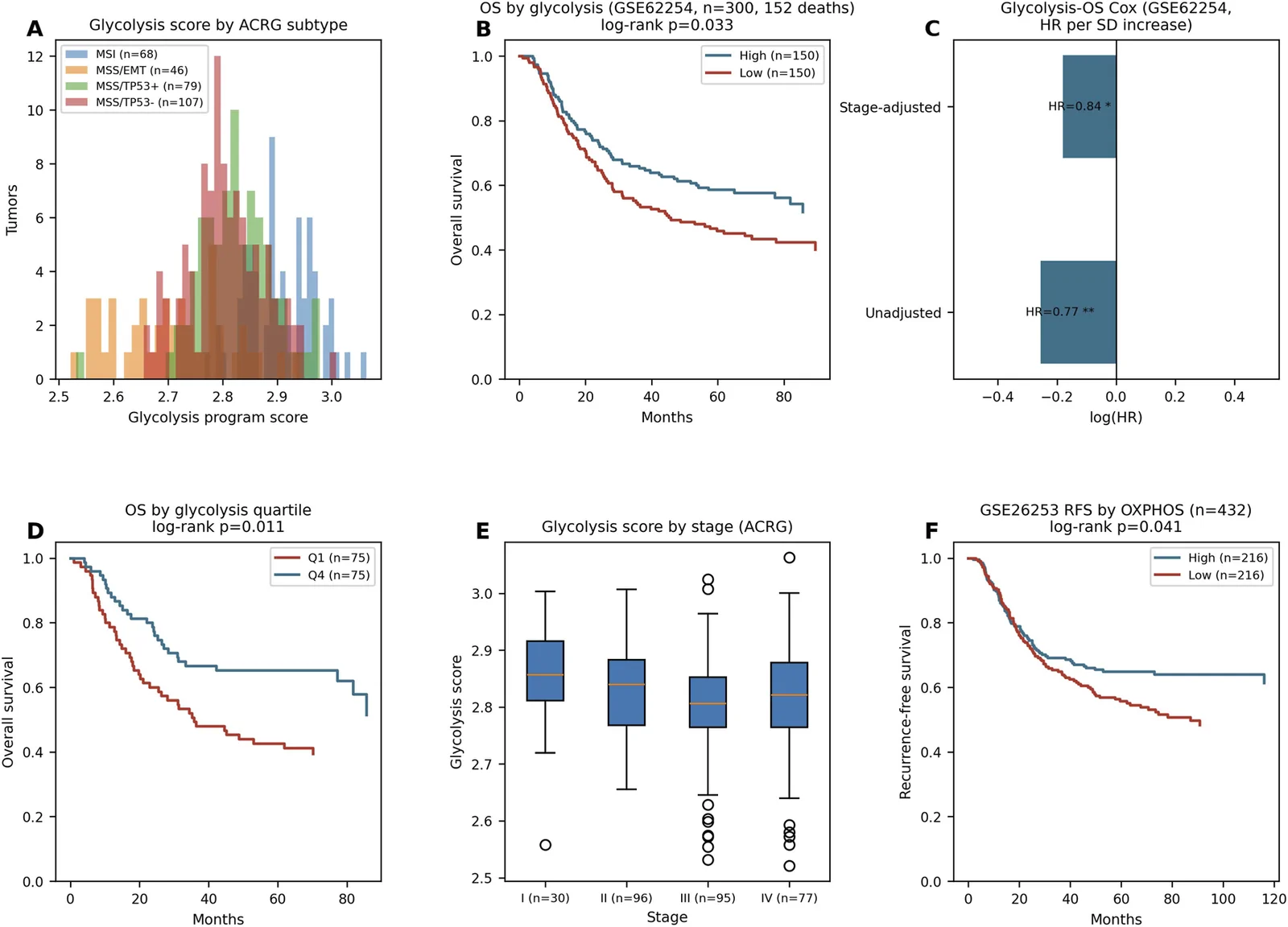
Prognostic associations of metabolic programs in independent gastric cancer cohorts. **(A)** Glycolysis program score distribution by ACRG molecular subtype (GSE62254; original coding 0 = MSS/TP53−, 1 = MSS/TP53+, 2 = MSI, 3 = MSS/EMT). **(B)** Kaplan–Meier overall survival by median glycolysis split in GSE62254 (verified coding: 152 deaths, 148 censored; log-rank *p* = 0.033). **(C)** Glycolysis-OS Cox HR per standard-deviation increase in GSE62254, unadjusted and stage-adjusted; HR < 1 denotes favorable prognosis; asterisks denote *p* < 0.05. **(D)** Kaplan–Meier by glycolysis quartiles (Q1 vs. Q4, log-rank *p* = 0.011). **(E)** Glycolysis score by pathologic stage (pStage I–IV) in GSE62254. **(F)** GSE26253 recurrence-free survival by median OXPHOS split (GEO coding 1 = recurrence; nominal log-rank *p* = 0.042, uncorrected).

### 3.6 Metabolic programs track molecular and clinical context

The glycolytic program varied strongly with molecular context in TCGA-STAD: MSI-H tumors expressed higher glycolysis than MSS tumors (Mann–Whitney *p* = 1.0e−9; Figure 6A), and glycolysis differed across Lauren classes (Kruskal–Wallis *p* = 2.5e−4; Figure 6E). Predicted oxaliplatin sensitivity was higher in stage III–IV than stage I–II tumors (Mann–Whitney *p* = 0.024; Figure 6B). The corresponding z-scored expression patterns of glycolytic genes in MSS and MSI-H tumors are shown in Figure 6C. Tumors in the high-glycolysis quartile were substantially enriched for the resistant-like group (chi-square test; Figure 6D). Consistent with their higher immune infiltration, MSI-H tumors showed elevated PD-1/PD-L1 axis expression (*p* = 3.3e−4; Figure 6F). Because MSI-H tumors carry both high glycolysis and a favorable prognosis, the MSI context plausibly reconciles the co-occurrence of inferred resistance markers with better survival observed in the validation cohorts.

**FIGURE 6:**
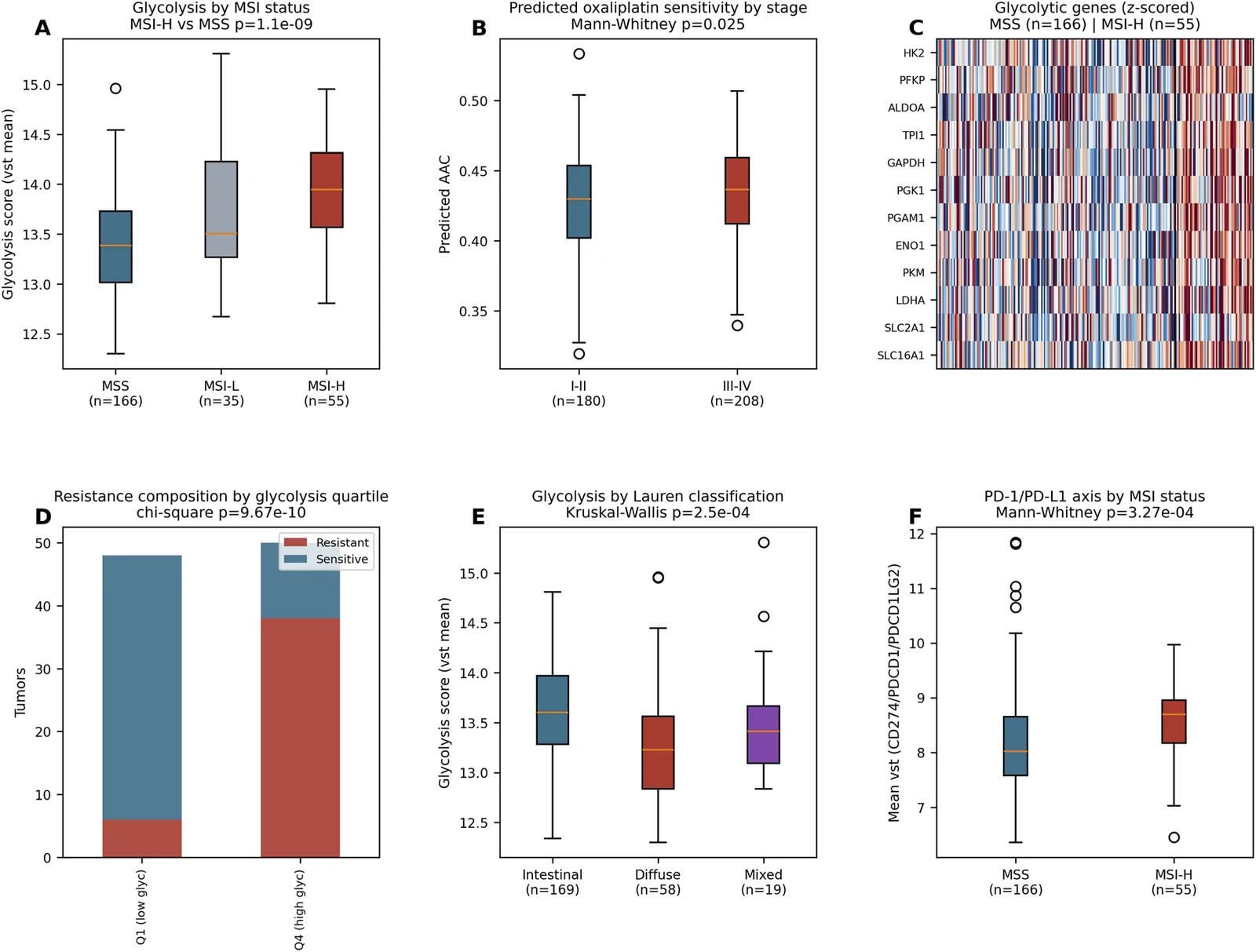
Molecular and clinicopathologic context of glycolysis and inferred oxaliplatin sensitivity. **(A)** Glycolysis program score by MSI status in TCGA-STAD (MSI-H vs. MSS, Mann–Whitney *p* = 1.0e-9). **(B)** Predicted oxaliplatin sensitivity by stage group I–II vs. III–IV (Mann–Whitney *p* = 0.024). **(C)** Glycolytic gene expression (z-scored) in MSS vs. MSI-H tumors; vertical line separates groups. **(D)** Resistant-like/sensitive-like composition across glycolysis quartiles (Q1 = lowest, Q4 = highest glycolysis; chi-square test). **(E)** Glycolysis score by Lauren classification (Kruskal–Wallis *p* = 2.5e−4). **(F)** PD-1/PD-L1 axis expression by MSI status (Mann–Whitney *p* = 3.3e−4).

### 3.7 Single-cell data localize the glycolytic program (descriptive)

Across 43,992 quality-filtered cells from 13 gastric biopsies (GSE134520), marker-based annotation identified epithelial cells as the dominant compartment (36,714 cells), with B/plasma, T-cell, stromal, and myeloid populations (Figures 7A–C). The glycolysis program was broadly expressed across the major cell types on UMAP (Figure 7D), with the highest median score in stromal cells (0.552), followed by epithelial cells (0.514; Figures 7E, 8C); epithelial cells, being the dominant compartment, contribute the largest share of glycolytic signal. At the sample level, the single early gastric cancer biopsy showed a higher epithelial glycolysis score than the three non-atrophic gastritis biopsies, but this contrast is descriptive only (EGC n = 1; no statistical inference is performed; Figures 7F, 8B).

**FIGURE 7:**
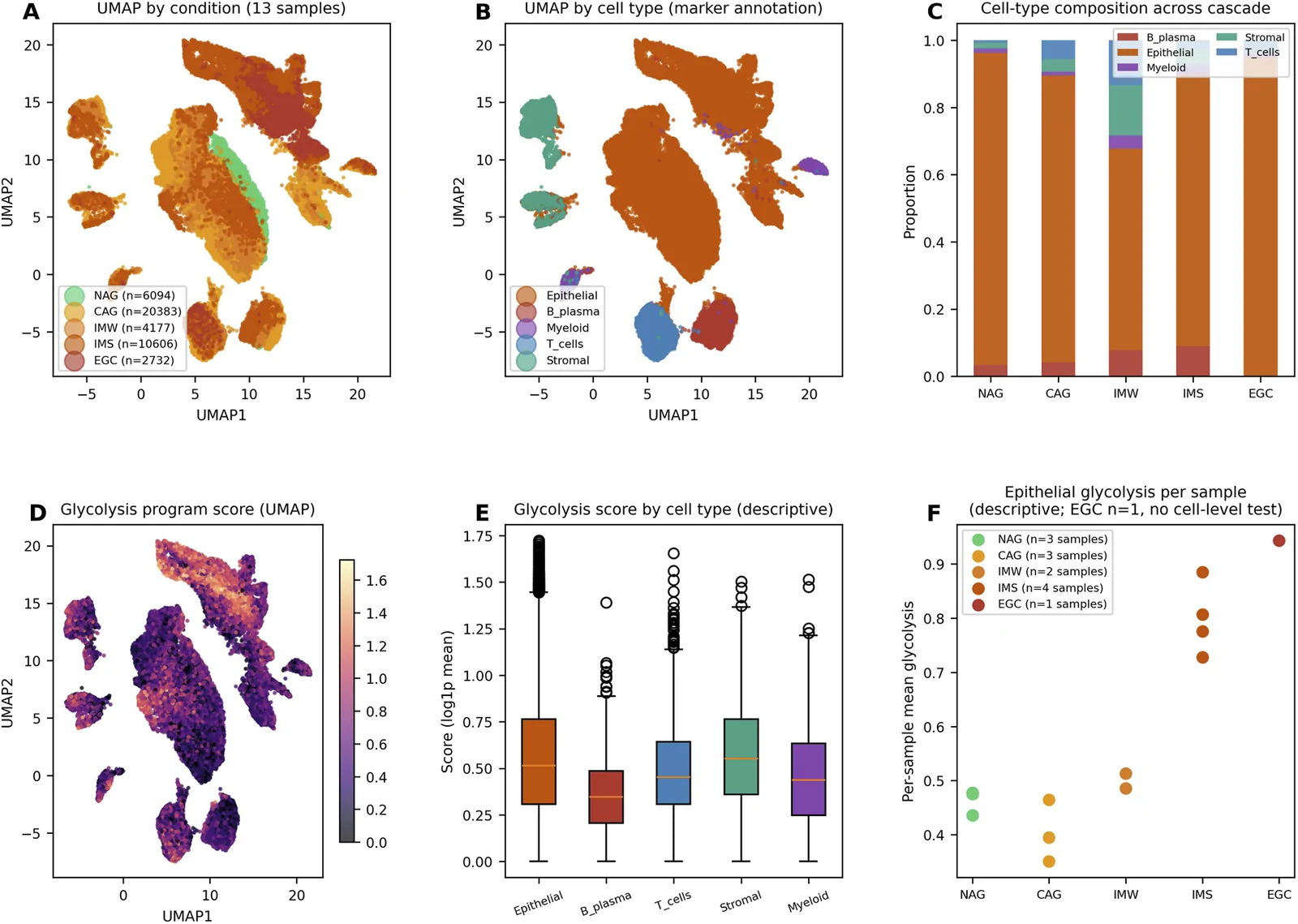
Single-cell localization of glycolytic activity across gastric cell populations. **(A)** UMAP of 43,992 cells colored by condition (NAG/CAG/IMW/IMS/EGC). **(B)** UMAP colored by marker-based cell type annotation. **(C)** Cell-type composition across the carcinogenesis cascade. **(D)** UMAP colored by glycolysis program score. **(E)** Glycolysis score by cell type (descriptive box plots; highest median in stromal cells). **(F)** Epithelial glycolysis at the sample level (13 biopsy means; descriptive only—EGC n = 1, no statistical test).

**FIGURE 8:**
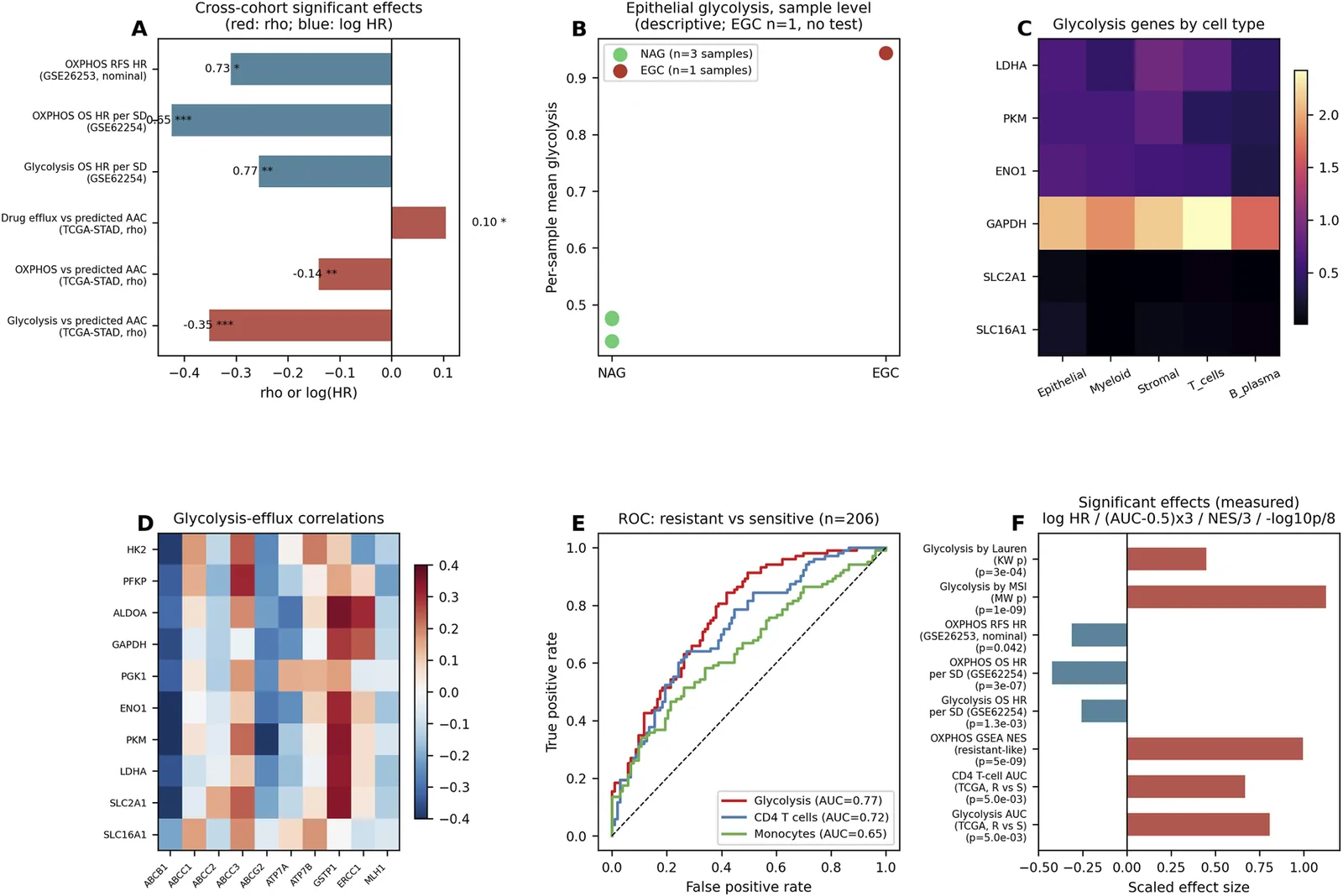
Integrated cross-cohort and single-cell summary of resistance-associated programs. **(A)** Cross-cohort significant effects: red bars = Spearman rho with predicted AAC (TCGA-STAD); blue bars = log Cox HR (GSE62254 HR per standard-deviation increase; GSE26253 HR high vs. low, nominal); only *p* < 0.05 shown; HR and p pairs are from the same models. **(B)** Epithelial glycolysis at the sample level in EGC (n = 1) versus NAG (n = 3) biopsies (descriptive, no test). **(C)** Mean expression of glycolysis genes across cell types in the single-cell atlas. **(D)** Spearman correlation heatmap between glycolysis genes and drug-efflux genes in TCGA-STAD. **(E)** ROC curves of the top three discriminators (glycolysis, CD4 T cells, monocytes) for resistant-like versus sensitive-like tumors; directions corrected so AUC ≥ 0.5. **(F)** Summary bar chart of the significant effects measured in this study; sizes use per-metric scaling: log HR, (AUC − 0.5) × 3, NES/3, or −log10(p)/8.

### 3.8 Integration and scope of evidence

Integrating the measured effects (Figure 8A), the glycolytic program associates with GDSC2-inferred oxaliplatin resistance in TCGA-STAD (rho = −0.35, *p* = 2.0e−13; AUC = 0.77), co-occurs with oxidative metabolism and reduced adaptive immune scores (Figures 8E,F), and is expressed across gastric cell types in the single-cell atlas. In two independent gastric cancer cohorts, these programs are favorably, not adversely, prognostic (Figure 8A). Correlations between glycolysis genes and drug-efflux genes are summarized in Figure 8D. As a measured negative control, inferred resistance itself was not prognostic for overall survival in TCGA-STAD (HR = 0.90, *p* = 0.64). Because the resistant-like/sensitive-like grouping and the downstream analyses share the same expression matrix, these analyses describe the transcriptional features of the model output rather than providing independent resistance validation; no clinical oxaliplatin response data are available for any cohort in this study.

### 3.9 *In vitro* qRT-PCR validation of selected metabolic genes

*In vitro* qRT-PCR in AGS gastric cancer cells showed differential mRNA expression after 24 h oxaliplatin exposure (10–20 μM) relative to untreated AGS controls (Figures 9A,B): ALB decreased by 68% (*p* < 0.001), whereas LDHA increased by 282% (*p* < 0.001), FASN by 315% (*p* < 0.001), and CPT1A by 252% (*p* < 0.001). These results provide limited cell-based mRNA corroboration for selected metabolic transcripts associated with the computational analyses. Because qRT-PCR measures RNA abundance rather than protein expression or pathway activity, these findings should be interpreted as exploratory and do not establish protein-level expression, spatial localization, or a causal mechanism of oxaliplatin resistance.

**FIGURE 9:**
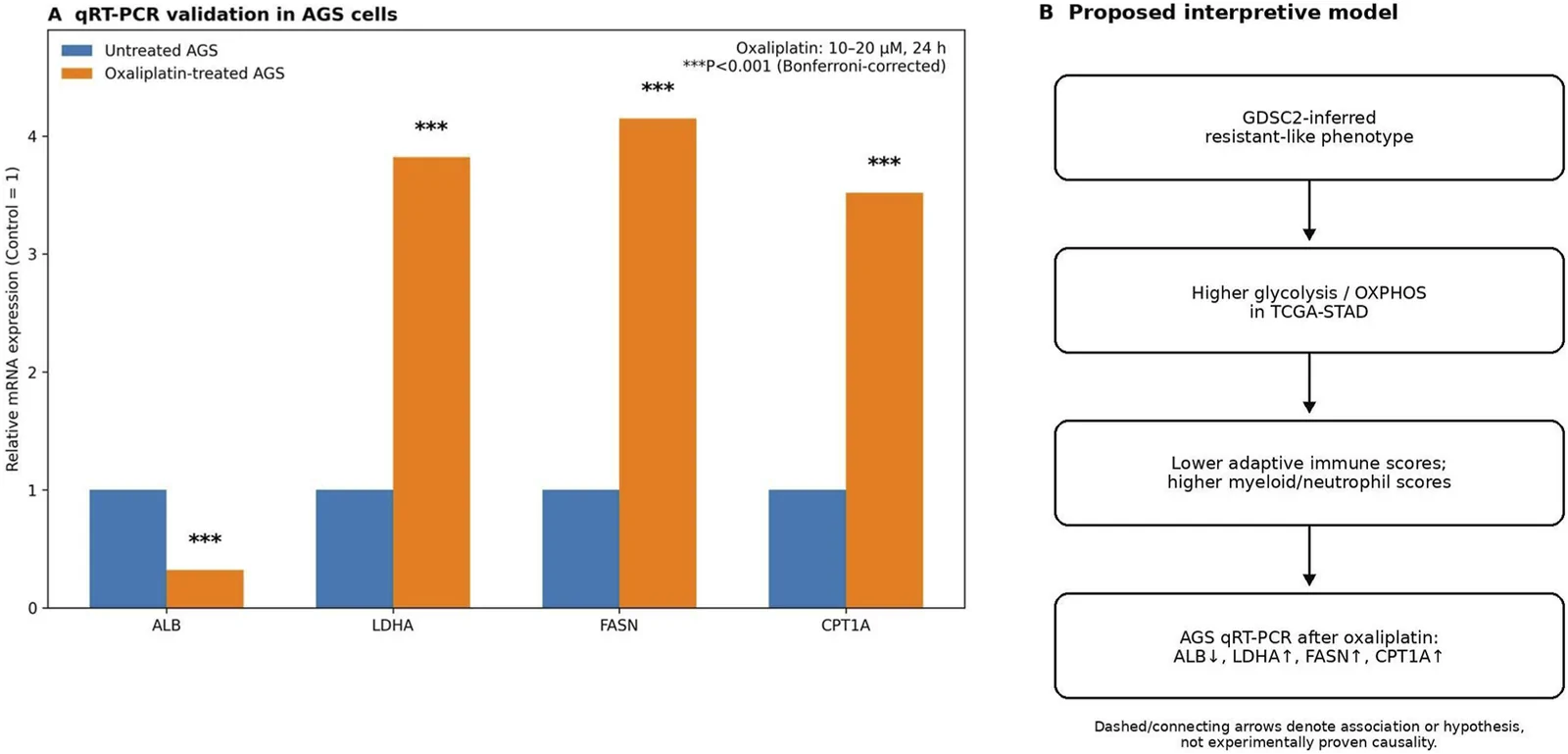
*In vitro* qRT-PCR validation in oxaliplatin-treated AGS cells and proposed interpretive model. **(A)** Relative mRNA expression of ALB, LDHA, FASN, and CPT1A in AGS gastric cancer cells after oxaliplatin exposure (10–20 μM, 24 h) compared with untreated AGS controls. Expression was normalized to GAPDH using the 2^−ΔΔCt^ method; reported significance for each gene was ***p < 0.001 after Bonferroni correction. qRT-PCR measures mRNA abundance and is presented as exploratory cell-based corroboration rather than protein-level or mechanistic validation. **(B)** Proposed literature-informed schematic summarizing associations among metabolic reprogramming, immune composition, and the GDSC2-inferred oxaliplatin-resistant-like phenotype. Arrows denote hypothesized or literature-supported relationships and should not be interpreted as mechanisms directly demonstrated by this study.

## 4 Discussion

This study integrates pharmacogenomic modeling, bulk transcriptomics, independent prognostic cohorts, single-cell data, and limited qRT-PCR validation to characterize molecular programs associated with GDSC2-inferred oxaliplatin sensitivity in GC. The principal finding is not a clinically validated resistance signature but a reproducible transcriptional pattern within the model output: resistant-like TCGA-STAD tumors showed higher glycolysis and oxidative phosphorylation activity, together with relatively reduced adaptive immune scores and increased myeloid/neutrophil scores. WGCNA additionally identified co-expression modules that tracked predicted AAC, while single-cell analysis localized glycolytic activity across epithelial, stromal, and immune compartments.

A central interpretive constraint is that the oxaliplatin phenotype is computationally inferred. GDSC is a valuable pharmacogenomic resource for linking molecular features to drug sensitivity (Iorio et al., 2016), but the present ridge model was trained across a pan-cancer cell-line set and transferred from an Affymetrix expression platform to TCGA RNA-seq data without cross-platform calibration. Moreover, resistant-like and sensitive-like tumors were defined from the same expression matrix subsequently used for downstream differential and pathway analyses. These analyses therefore describe transcriptional correlates of the model output rather than independent validation of drug resistance. This distinction is important because experimentally demonstrated oxaliplatin resistance in GC involves multiple mechanisms, including TOPBP1/PARP1-associated DNA-repair regulation and JUNB/MAPK signaling (Fang et al., 2022; Li et al., 2023).

The enrichment of glycolytic and oxidative programs in resistant-like tumors is biologically plausible but should not be interpreted as proof that these pathways cause resistance. Metabolic competition can shape tumor and immune-cell behavior, and lactate-rich environments can alter macrophage polarization and antitumor immunity (Chang et al., 2015; Colegio et al., 2014; Ippolito et al., 2019). In our data, the inferred resistant state was accompanied by higher myeloid/neutrophil scores and lower adaptive immune scores, supporting an association between metabolic state and immune composition. However, the marker-based immune scores are transcriptomic approximations, not direct measurements of cell abundance or function, and no perturbation experiment was performed to demonstrate causal metabolic-immune coupling.

An important result is that metabolic programs associated with inferred resistance were not consistently adverse prognostic markers. In GSE62254, higher glycolysis, oxidative phosphorylation, and carnitine/fatty-acid-oxidation program expression showed favorable or neutral survival associations, and in GSE26253, higher oxidative-phosphorylation expression was nominally associated with fewer recurrences. This apparent paradox is compatible with the molecular heterogeneity of GC described by TCGA and ACRG classifications (Li et al., 2023; Iorio et al., 2016). In the present analysis, MSI-H tumors exhibited higher glycolysis, and MSI status is itself linked to a distinct molecular and prognostic context. Thus, a transcriptional program that correlates with model-inferred drug sensitivity cannot be assumed to carry the same direction of association with untreated or heterogeneously treated survival outcomes.

The single-cell analysis provides localization rather than validation of drug response. GSE134520 was originally generated to map cellular changes across gastric premalignant lesions and early GC (Zhang et al., 2019). In our re-analysis, glycolysis scores were present across major cell types and were highest in stromal cells, while epithelial cells contributed the largest absolute share because they dominated the dataset. The early-GC condition contains a single biopsy, so condition-level comparisons involving EGC are descriptive only. These observations help identify cellular compartments in which the program is expressed but do not establish that any particular cell type mediates oxaliplatin resistance.

The qRT-PCR experiment similarly warrants conservative interpretation. In AGS gastric cancer cells exposed to oxaliplatin (10–20 μM, 24 h), ALB mRNA was lower, whereas LDHA, FASN, and CPT1A mRNA levels were higher than in untreated controls. The official gene symbol ALB is used throughout the revised manuscript to avoid confusion with the clinical albumin–bilirubin (ALBI) score. These measurements assess mRNA abundance only; they do not demonstrate corresponding protein changes or establish that these genes causally mediate oxaliplatin resistance. Independent concentration-response experiments, functional perturbation studies, and orthogonal protein-level assays would be required before these genes could be considered mechanistically validated determinants of oxaliplatin response.

## 5 Conclusion

This integrative analysis identifies metabolic and immune transcriptional features associated with GDSC2-inferred oxaliplatin sensitivity in GC, with glycolysis emerging as the strongest program-level discriminator in TCGA-STAD. The findings are intentionally framed as model-associated rather than clinically validated resistance biology. Independent prognostic cohorts showed that several resistance-associated metabolic programs were favorably or neutrally prognostic, highlighting the non-equivalence of pharmacogenomic sensitivity inference and survival. Single-cell analysis provided descriptive cell-type localization, and qRT-PCR in oxaliplatin-treated AGS cells offered limited mRNA-level corroboration for selected metabolic genes. Direct validation in independent, clinically annotated oxaliplatin-treated cohorts, together with additional dose-response, functional, and protein-level studies, is required before these associations can support predictive or mechanistic claims.

## Data Availability

TCGA-STAD data are available from the NCI Genomic Data Commons. GSE62254, GSE26253, and GSE134520 are available from the NCBI Gene Expression Omnibus. GDSC2 pharmacogenomic data were obtained from the ORCESTRA PharmacoSet resource (v2-8.2), as described in the Methods.

## References

[B1] Cancer Genome Atlas Research Network (2014). Comprehensive molecular characterization of gastric adenocarcinoma. Nature 513 (7517), 202–209. 10.1038/nature13480 25079317 PMC4170219

[B2] Chang C. H. Qiu J. O'Sullivan D. Buck M. D. Noguchi T. Curtis J. D. (2015). Metabolic competition in the tumor microenvironment is a driver of cancer progression. Cell 162 (6), 1229–1241. 10.1016/j.cell.2015.08.016 26321679 PMC4864363

[B3] Colegio O. R. Chu N. Q. Szabo A. L. Chu T. Rhebergen A. M. Jairam V. (2014). Functional polarization of tumour-associated macrophages by tumour-derived lactic acid. Nature 513 (7519), 559–563. 10.1038/nature13490 25043024 PMC4301845

[B4] Cristescu R. Lee J. Nebozhyn M. Kim K. M. Ting J. C. Wong S. S. (2015). Molecular analysis of gastric cancer identifies subtypes associated with distinct clinical outcomes. Nat. Med. 21 (5), 449–456. 10.1038/nm.3850 25894828

[B5] Fang Z. Gong C. Ye Z. Wang W. Zhu M. Hu Y. (2022). TOPBP1 regulates resistance of gastric cancer to oxaliplatin by promoting transcription of PARP1. DNA Repair (Amst) 111, 103278. 10.1016/j.dnarep.2022.103278 35124372

[B6] Iorio F. Knijnenburg T. A. Vis D. J. Bignell G. R. Menden M. P. Schubert M. (2016). A landscape of pharmacogenomic interactions in cancer. Cell 166 (3), 740–754. 10.1016/j.cell.2016.06.017 27397505 PMC4967469

[B7] Ippolito L. Morandi A. Giannoni E. Chiarugi P. (2019). Lactate: a metabolic driver in the tumour landscape. Trends Biochem. Sci. 44 (2), 153–166. 10.1016/j.tibs.2018.10.011 30473428

[B8] Langfelder P. Horvath S. (2008). WGCNA: an R package for weighted correlation network analysis. BMC Bioinforma. 9, 559. 10.1186/1471-2105-9-559 19114008 PMC2631488

[B9] Lee J. Sohn I. Do I. G. Kim K. M. Park S. H. Park J. O. (2014). Nanostring-based multigene assay to predict recurrence for gastric cancer patients after surgery. PLoS One 9 (3), e90133. 10.1371/journal.pone.0090133 24598828 PMC3943911

[B10] Li S. Wei Y. Sun X. Liu M. Zhu M. Yuan Y. (2023). JUNB mediates oxaliplatin resistance *via* the MAPK signaling pathway in gastric cancer by chromatin accessibility and transcriptomic analysis. Acta Biochim. Biophys. Sin. (Shanghai) 55 (11), 1784–1796. 10.3724/abbs.2023119 37337631 PMC10679881

[B11] Love M. I. Huber W. Anders S. (2014). Moderated estimation of fold change and dispersion for RNA-seq data with DESeq2. Genome Biol. 15 (12), 550. 10.1186/s13059-014-0550-8 25516281 PMC4302049

[B12] Newman A. M. Liu C. L. Green M. R. Gentles A. J. Feng W. Xu Y. (2015). Robust enumeration of cell subsets from tissue expression profiles. Nat. Methods 12 (5), 453–457. 10.1038/nmeth.3337 25822800 PMC4739640

[B13] Ritchie M. E. Phipson B. Wu D. Hu Y. Law C. W. Shi W. (2015). Limma powers differential expression analyses for RNA-sequencing and microarray studies. Nucleic Acids Res. 43 (7), e47. 10.1093/nar/gkv007 25605792 PMC4402510

[B14] Smyth E. C. Nilsson M. Grabsch H. I. van Grieken N. C. T. Lordick F. (2020). Gastric cancer. Lancet 396 (10251), 635–648. 10.1016/S0140-6736(20)31288-5 32861308

[B15] Subramanian A. Tamayo P. Mootha V. K. Mukherjee S. Ebert B. L. Gillette M. A. (2005). Gene set enrichment analysis: a knowledge-based approach for interpreting genome-wide expression profiles. Proc. Natl. Acad. Sci. U. S. A. 102 (43), 15545–15550. 10.1073/pnas.0506580102 16199517 PMC1239896

[B16] Sung H. Ferlay J. Siegel R. L. Laversanne M. Soerjomataram I. Jemal A. (2021). Global cancer statistics 2020: GLOBOCAN estimates of incidence and mortality worldwide for 36 cancers in 185 countries. CA Cancer J. Clin. 71 (3), 209–249. 10.3322/caac.21660 33538338

[B17] Wolf F. A. Angerer P. Theis F. J. (2018). SCANPY: large-scale single-cell gene expression data analysis. Genome Biol. 19 (1), 15. 10.1186/s13059-017-1382-0 29409532 PMC5802054

[B18] Yu G. Wang L. G. Han Y. He Q. Y. (2012). clusterProfiler: an R package for comparing biological themes among gene clusters. OMICS 16 (5), 284–287. 10.1089/omi.2011.0118 22455463 PMC3339379

[B19] Zhang P. Yang M. Zhang Y. Xiao S. Lai X. Tan A. (2019). Dissecting the single-cell transcriptome network underlying gastric premalignant lesions and early gastric cancer. Cell Rep. 27 (6), 1934–1947.e5. 10.1016/j.celrep.2019.04.052 31067475

